# 3D Printing for Customized Bone Reconstruction in Spheno-Orbital Meningiomas: A Systematic Literature Review and Institutional Experience

**DOI:** 10.3390/jcm13133968

**Published:** 2024-07-06

**Authors:** Simona Serioli, Alberto Pietrantoni, Alberto Benato, Marco Galeazzi, Amedeo Piazza, Liverana Lauretti, Pier Paolo Mattogno, Alessandro Olivi, Marco Maria Fontanella, Francesco Doglietto

**Affiliations:** 1 Division of Neurosurgery, Department of Medical and Surgical Specialties, Radiological Sciences, and Public Health, Spedali Civili of Brescia, University of Brescia, 25123 Brescia, Italy; marco.fontanella@unibs.it; 2 Department of Neurosurgery, Fondazione Policlinico Universitario Agostino Gemelli IRCCS, 00168 Rome, Italy; benato.alberto@gmail.com (A.B.); mgaleazzi@live.it (M.G.); liverana.lauretti@policlinicogemelli.it (L.L.); pierpaolo.mattogno@policlinicogemelli.it (P.P.M.); alessandro.olivi@policlinicogemelli.it (A.O.); 3 Pathology Unit, Spedali Civili of Brescia, University of Brescia, 25123 Brescia, Italy; alpietra94@gmail.com; 4 Neurosurgery Division, Department of Neuroscience, “Sapienza” University of Rome, 00185 Rome, Italy; amedeo.piazza@uniroma1.it; 5 Neurosurgery, School of Medicine, Università Cattolica del Sacro Cuore, 00168 Rome, Italy

**Keywords:** custom cranial implant, customized reconstruction, skull base, spheno-orbital meningiomas, spheno-orbital reconstruction, 3D printing customized prosthesis

## Abstract

**Background**: The treatment of spheno-orbital meningiomas (SOMs) requires extensive bone resections, creating significant defects in a complex geometrical space. Bone reconstruction represents a fundamental step that optimizes long-term aesthetic and functional outcomes. In recent years, 3D printing technology has also been exploited for complex skull base reconstructions, but reports remain scarce. **Methods**: We retrospectively analyzed four consecutive patients who underwent SOM resection and one-step 3D PEEK customized reconstruction from 2019 to 2023. A systematic review of 3D printing customized implants for SOM was then performed. **Results**: All patients underwent a frontotemporal craniotomy, removal of SOM, and reconstruction of the superolateral orbital wall and pterional region. The aesthetic outcome was extremely satisfactory in all cases. No orbital implant malposition or infectious complications were documented. Eleven papers were included in the literature review, describing 27 patients. Most (23) patients underwent a single-stage reconstruction; in three cases, the implant was positioned to correct postoperative delayed enophthalmos. Porous titanium was the most used material (16 patients), while PEEK was used in three cases. Prosthesis malposition was described in two (7.4%) patients. **Conclusions**: Single-step reconstruction with a personalized 3D PEEK prosthesis represents a valid reconstruction technique for the treatment of SOMs with good aesthetic outcomes.

## 1. Introduction

Spheno-orbital meningiomas (SOM) pose significant challenges due to their characteristic features, which usually include a pronounced hyperostotic reaction in the superior and lateral orbital walls, as well as the pterional calvarium. Surgical management typically involves a resection of the affected bone, resulting in notable structural defects, which are geometrically complex and might have an effect both on the functional anatomy and aesthetics of the patient [1,2]. The reconstruction represents a complex procedure that may be performed in one or more steps [3], with different options being described in the literature [1,4]. Traditionally, approaches to address the bony defects have varied widely, from the use of autologous bone graft (calvaria inner table, iliac bone, or costal graft) [5,6,7], to several materials such as titanium mesh, porous polyethylene, methylmethacrylate, and polymethylmethacrylate, which are modeled during the reconstruction phase [4,6,8,9,10].

Technological advancements have recently introduced patient-specific, pre-designed implants. By leveraging 3D printing technology, prostheses tailored to fit the anticipated bone resection and replace the hyperostotic bone can be created [11]. These innovative techniques require reconstruction materials, such as printed CAD/CAM titanium [3,12,13,14,15,16], polyether ether ketone (PEEK) [17,18], and polymethylmethacrylate (PMMA) [11,13,19,20]. They might offer several advantages, including streamlining the surgical procedure and enhancing aesthetic outcomes through meticulous planning and design. Despite the potential advantages, the experiences reported in the literature with the use of custom-made implants for skull reconstruction in spheno-orbital meningiomas are limited.

This study aimed to share our experience with custom-made PEEK implants for one-step reconstruction of the pterion and orbit after the removal of meningo-orbital meningiomas. Additionally, a systematic review, according to the Preferred Reporting Items for Systematic Reviews and Meta-Analyses (PRISMA) guidelines [21], on 3D custom-made cranioplasty in the management of spheno-orbital meningiomas is provided.

## 2. Materials and Methods

### 2.1. Search Methods 

A systematic review based on the PRISMA guidelines was performed [21]. The electronic databases PubMed, Scopus, and Web of Science were investigated on February 1, 2024. The following keywords and their combinations were searched: “sphenoorbital”, “spheno-orbital”, “reconstructi*”, “implant”,” prosthesis”, “custom-made”, “custom made”, and “patient specific”. The results of the database searches were analyzed using Zotero software (version 6.0.37). After the manual removal of duplicates, the analysis of the title and the selection of full text were performed by two independent researchers (S.S. and A.B.). In case of doubts or disagreement, the supervision of a senior author (F.D.) was requested.

### 2.2. Selection Criteria and Data Extraction

All the studies describing customized 3D-printed reconstructions after surgical excision of spheno-orbital meningiomas were included. The exclusion criteria were the following: (1) reviews of the literature; (2) clinical studies without reconstruction or (3) 3D printing; (4) other pathologies than spheno-orbital meningiomas; (5) papers with incomplete data or (6) written in a language other than English; (7) cadaveric or postmortem studies; (8) conference abstracts, and comments. 

One of the authors (A.B.) extracted data from the articles, which were then verified and supervised by another author (S.S.). In case of disagreement, a senior author (F.D.) analyzed the information independently to reach a consensus.

For each study, the authors extracted the following information: (1) first author and year of publication, (2) age, (3) gender, (4) reconstruction material, (5) surgical approach, (6) steps of reconstruction, (7) pre-operative symptoms, (8) grade of surgical resection (9) post-operative neurology, (10) complications, (11) clinical outcome (mRS) with mean follow up, (12) cosmetic outcome, and (13) adjuvant radiotherapy. Where reported, the cosmetic outcomes were numerically classified from one to five according to the following scale: (1) no asymmetry of the face and temporal region; (2) asymmetry without deformity; (3) asymmetry with slight deformity; (4) asymmetry with significant deformity; and (5) disfiguring asymmetry.

### 2.3. Clinical Study

A retrospective and consecutive cohort of patients treated from 2019 to 2023 with a diagnosis of spheno-orbital meningioma, who underwent surgical resection followed by one-step 3D-printed customized reconstruction, was collected. The patients’ records, or visits were collected (S.S., A.P. (Alberto Pietrantoni), and M.G.) to obtain the latest clinical follow-up. PEEK prostheses (RecranioTM, Medprin Biotech GmbH, Frankfurt am Main, Germany) were used in all cases. 

Clinical data, including age, sex, previous surgery, preoperative symptoms, type of resection, postoperative symptoms and complications, WHO-grade tumor, and duration of follow-up were reviewed. A fronto-temporal craniotomy with neuronavigation support was performed in all cases; furthermore, intraoperative neuromonitoring was used in two cases.

A pre-operative CT scan was performed for each patient, with a thickness of 0.625 mm. Starting from the DICOM (Digital Imaging and Communications in Medicine) data, the virtual model of the prosthesis was realized from the patient’s anatomy, carefully identifying the areas of bony infiltration. By creating an interactive 3D PDF file (Acrobat Reader, Adobe, San Jose, CA, USA), the prosthesis was sized by the senior neurosurgeon (F.D.) according to the extension of the defect caused by the tumor while considering a safety margin of one centimeter from the ideal limit of the planned craniotomy. Moreover, a cutting guide was created to support the surgeon during the craniotomy. After the final validation, the implant was produced through a laser powder-sintering process.

Before implantation, the customized prosthesis required sterilization at 134 °C for four minutes with a pressure reference range of between 205 and 230 kpa.

The patients’ informed consent was obtained according to the institutional guidelines, and the study was conducted according to the Declaration of Helsinki. 

The descriptive statistical analysis was performed using the software Jamovi Version 2 statistic software (The jamovi project (2022) (Version 2.3) [Computer Software, Sidney—https://www.jamovi.org/ accessed on 5 April 2024]. All numerical data are standardized to the first decimal place after the point.

## 3. Results

### 3.1. Review of the Literature

#### 3.1.1. Study Selection

**Figure 1:**
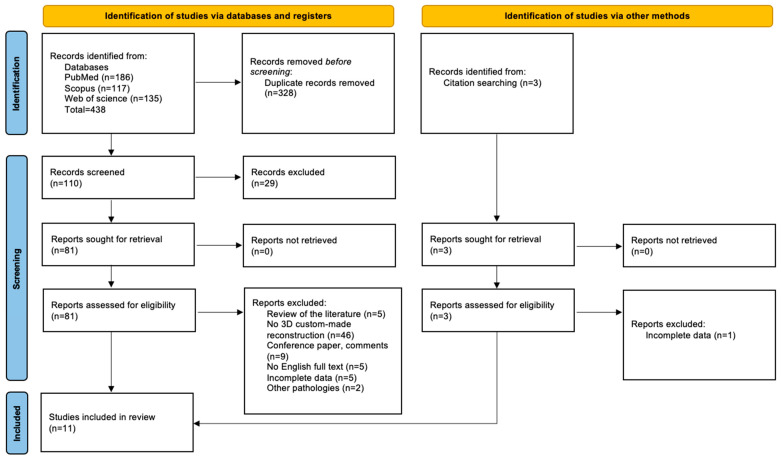
PRISMA flowchart.

The search on the electronic databases (PubMed, Scopus, and Web of Science) yielded 438 results. Three additional papers were manually included after analyzing the citations. After removing 328 duplicates, 29 papers were excluded by analyzing the title and abstract. A total of 81 studies were selected for the full-text phase, and 11 papers were included in the results. The PRISMA flow chart is shown in Figure 1.

#### 3.1.2. Summary of Results

All studies included in the systematic review were retrospective; they were published from 2009 to 2023, totaling eleven articles and 27 patients [3,11,12,13,14,15,16,17,18,19,20]. Table 1 summarizes the reported data.

The age at surgery ranged from 33 to 89 years (mean 52.5 years, SD ± 11.9), and a female predominance was evident (77.8%, 21 patients). The most common presenting symptoms were exophthalmos (51.9%, 14 patients), diplopia (37%, 10 patients), visual impairment (18.5%, 5 patients), and orbital dystopia (18.5%, 5 patients), while enophthalmos was evident in 3 patients (11.1%) who underwent delayed reconstruction. 

Among the different materials, porous titanium was used in 16 patients (59.3%), polymethylmethacrylate (PMMA) in 8 patients (29.6%), and PEEK in 3 patients (11.1%). In most (20; 74%) patients, both the pterional and orbital regions were reconstructed by a single prosthesis; in one series, only the fronto-temporal region was reconstructed; and in another series of seven patients, two separate titanium prostheses were used to reconstruct the orbit and the pterion [15]. 

In four cases (14.8%), the reconstruction was performed in two steps (mean of 19.3 months after initial surgery, range of 12–24 months) [3,14]; in all the other patients, tumor removal and skull reconstruction were performed in a single step (85.2%). Regarding the surgical approach, the fronto-temporal craniotomy was the most used (22 patients, 81.5%), followed by the subciliary (used in 2 patients, 7.4%, at the second stage), transpalpebral (1 patients, 3.7%) and combined approach (1 patient with an endoscopic transorbital approach and 1 patient with a complex, second stage reconstruction after orbital exenteration). 

Postoperatively, five patients developed diplopia (18.5%), which was transient in three cases, and one palpebral ptosis. Enophthalmos was identified in one case after the transpalpebral surgery. Prosthesis malposition with the necessity of surgical revision was described in two patients (7.4%) by two different groups, while an epidural fluid collection affected one patient but was treated conservatively. No case of infection was described.

A comprehensive assessment of cosmetic outcomes was provided in 14 patients detailed across seven publications, either through postoperative photographs or detailed descriptions. Among these cases, eight patients exhibited excellent outcomes with no visible facial or temporal asymmetry. Four patients displayed minor asymmetries without notable deformity, while two patients presented with significant facial asymmetry accompanied by notable deformity.

The mean follow-up was between 1.2 and 36 months. Four patients required adjuvant radiotherapy (14.8%).

### 3.2. Institutional Case Series

The institutional case series includes four patients, three females and one male, with an average age of 58.5 years (range: 53–63 years) (Table 2). The mainly documented clinical signs and symptoms were exophthalmos (100%, four patients), diplopia (50%, two patients), swelling in the temporal region (50%, two patients), visual impairment (50%, two patients), and conjunctival hyperemia (25%, one patient). Oculomotor cranial nerve deficit was reported in one patient, who had ptosis and exotropia. No endocrine dysfunctions were reported.

One patient had undergone surgery 19 and 10 years prior to tumor recurrence removal and reconstruction. Hyperostosis at the level of the greater sphenoid wing was the common radiological feature, especially of the superior lateral wall of the orbit. The tumoral involvement of the anterior clinoid process was identified in one patient.

Intraoperative neuromonitoring (electroencephalography (EEG), motor evoked potential (MEP), somatosensory evoked potential (SSEP), and electromyography (EMG) of cranial nerves) was mandatory in two cases, considering the invasive features at the level of the superior orbital fissure and middle fossa.

A fronto-temporal craniotomy with excision of the hyperostotic bone was performed in all cases, and the removal of the lateral orbital rim in two.

Near total resection was achieved in two cases, and subtotal resection (STR) was performed in the remaining two. In all four cases, the histological diagnosis was meningothelial meningioma (Grade I), according to the World Health Organization Classification of Tumors of the Central Nervous System [22], with a low mitotic count (0–1 mitotic figures every ten high power fields) and Ki-67 labeling index at almost 4–5%. P53 immunohistochemical expression was available in only one case, and it was positive in 40% of neoplastic cell nuclei.

As for post-surgery complications, one patient developed transient anterior blepharitis. Two patients had transient cranial nerve deficits, namely paralysis of the temporal branch of the facial nerve, and of the trigeminal nerve (V2 branch). Exophthalmos was completely resolved or significantly improved in all patients; enophthalmos was not evident in any patient. During radiological follow-up (mean: 25; range: 9–41), the tumoral residuals remained stable, so no further adjuvant radiotherapy treatments have been planned. Regarding the aesthetic outcome, all the patients are fully satisfied with the final aesthetic result, obtaining the maximum score in the dedicated score.

#### 3.2.1. Case Example 1

A 53-year-old Caucasian woman (patient 4 in Table 2) presented with progressive right eye proptosis and swelling in the temporal region, associated with visual impairment and diplopia due to dysfunction of the lateral rectus muscle.

**Figure 2:**
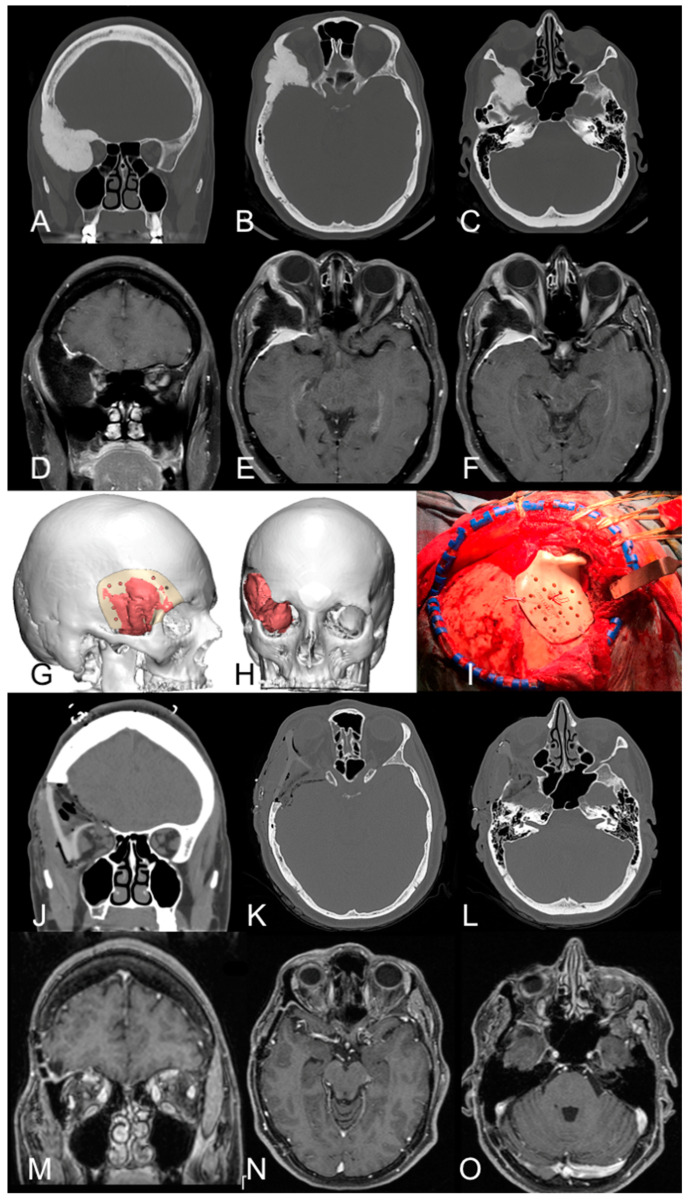
Pre- and postoperative neuroradiology; surgical planning and intraoperative image. (**A**–**F**). Coronal (**A**), and axial (**B**,**C**) CT scans and brain MRI T1-weighted sequences with contrast (coronal—(**D**); axial—(**E**,**F**)) show the right spheno-orbital meningioma with the typical features of hyperostosis at the level of the superior and lateral walls of the orbit, and sphenoid wing, with the tumor-induced remodeling of the middle cranial fossa. The lesion caused compression of the orbital cavity with associated exophthalmos. (**G**,**H**) Three-dimensional digital reconstruction of the tumor (in red) and the skull prosthesis based on the patient’s anatomy and tumor extension. (**I**) Intraoperative image of the prosthesis positioned at the end of the tumor removal with the reconstruction of the pterional region and orbital rim. (**J**–**O**) Postoperative coronal (**J**) and axial (**K**,**L**) CT scans and brain MRI three years after surgery (coronal—(**M**); axial—(**N**,**O**)) show complete removal of the lesion and optimal positioning of the prosthesis. The resolution of the pre-operative exophthalmos is also shown.

A brain MRI with gadolinium documented a right spheno-orbital meningioma with significant hyperostosis of the frontal and sphenoid bones, as well as the superior and lateral walls of the orbit. The tumor extended under the temporalis muscle and spread at the level of the middle skull base, involving the superior orbital fissure and foramen rotundum (Figure 2A–F).

The patient underwent a right fronto-temporal craniotomy with a one-step custom-made reconstruction (Figure 2G,H), with the support of intraoperative neuromonitoring (EEG, MEP, SSEP, and EMG of right CN III). A pericranial flap was harvested, and interfascial dissection was performed. After dissection of the exophytic portion of the lesion from the temporalis muscle, the area of bony hyperostosis was carefully resected. After dissection and cutting of the meningo-orbital fold, the orbit, the superior orbital fissure, and the second trigeminal branch were completely decompressed using an ultrasonic aspirator to remove the hyperostotic bone close to the cranial nerves. The lateral orbital rim was removed and subsequently reconstructed with the prosthesis.

The intradural component of the meningioma, which was adherent to the arachnoid at the level of the Silvian fissure, was completely removed, respecting the arachnoid plane and coagulating the vascular supply. The intraoperative stimulation of the third cranial nerve (tEMG) resulted in a prompt muscular response.

After dural reconstruction with the pericranium (the remaining flap was used to cover the partially opened frontal sinus) and using epidural periumbilical fat to fill empty spaces at the epidural level, the custom-made operculum was positioned at the level of the craniectomy and the partial orbital rim removal that had been previously performed, exploiting the cutting guide (Figure 2I). The operculum, which included a reconstruction of the superior and lateral walls of the orbit, was fixed with plates and screws; positioning was relatively straightforward and appeared satisfactory intraoperatively.

In the postoperative period, an incomplete right oculomotor nerve deficit occurred, with diplopia and palpebral ptosis, and a transient hypoesthesia in V2 distribution. The CT scan showed the optimal positioning of the implant without epidural blood collection (Figure 2J–L). Histological examination established the diagnosis of CNS WHO grade I meningioma, characterized by extensive infiltration of surrounding bony tissues and low proliferative index (Ki67 2–3%).

Five months after surgery, the exophthalmos had completely regressed. At the latest follow-up, the vision has significantly improved, while diplopia persists in the extreme position of the gauze. The follow-up MRI after three years documented the macroscopically complete excision of the tumor, with no signs of recurrence (Figure 2M–O).

#### 3.2.2. Case Example 2

A 60-year-old Caucasian male (patient 1 in Table 2) presented with progressive left eyelid ptosis with increasing temporal swelling, left eye exotropia, dystopia, and diplopia.

**Figure 3:**
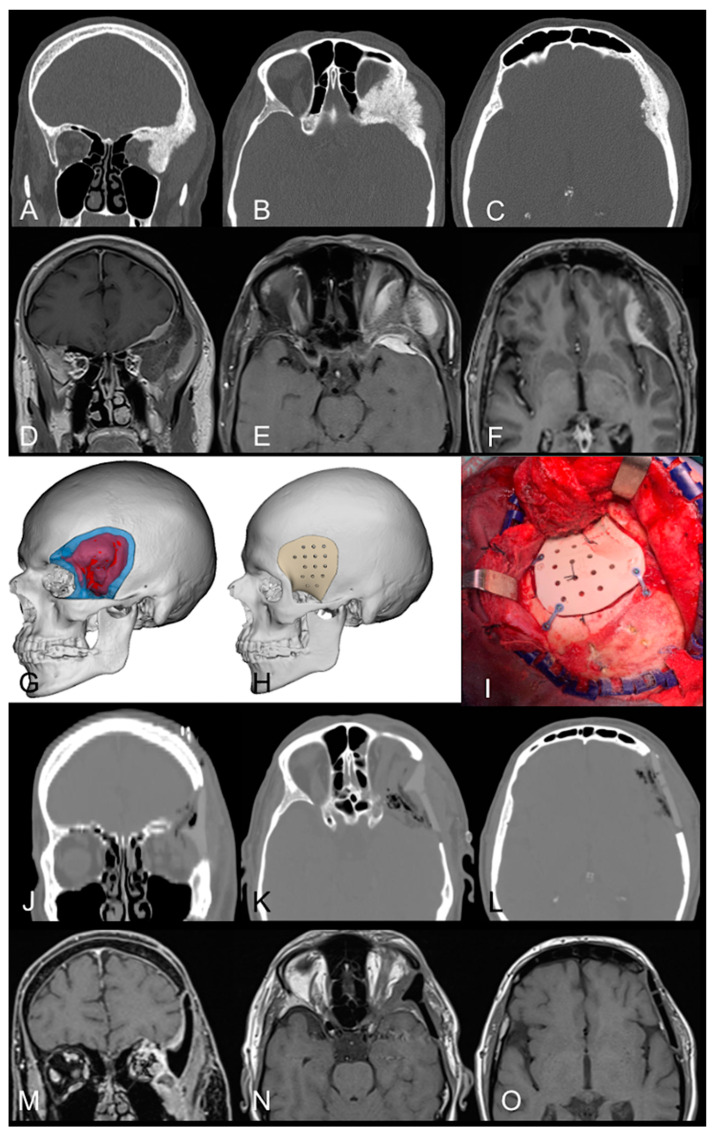
Pre- and postoperative neuroradiology, surgical planning with cutting guide, and intraoperative image. (**A**–**F**). Coronal (**A**), and axial (**B**,**C**) CT scans and brain MRI T1-weighted sequence with contrast (coronal—(**D**); axial—(**E**,**F**)) document the left spheno-orbital meningioma with hyperostosis at the level of the superior and lateral walls of the orbit, as well as the frontal bone in the pterional region. The lesion causes significant mass effect on the lateral and superior rectus muscle with exophthalmos and orbital dystopia. (**G**,**H**) Three-dimensional digital reconstruction of the CT of the patient, highlighting the extension of the tumor (in red) and the custom-made prosthesis (**H**). (**I**) Intraoperative image of the positioning of the implant with the reconstruction of the pterion. (**J**–**O**) Immediate postoperative CT scan with coronal (**J**) and axial (**K**,**L**) images. Brain MRI with contrast at four months after surgery (coronal—(**M**); axial—(**N**,**O**)) documents resolution of exophthalmos and temporal swelling and shows a residue into the orbit in tight contact with the lateral rectus muscle.

A CT scan and brain MRI documented a left spheno-orbital meningioma characterized by an extension to the ipsilateral frontal and sphenoid bone, with a mass effect on the lateral and superior rectus muscles and on the structures of the orbital apex, which appeared compressed and dislocated medially, resulting in proptosis of the left eye (Figure 3A–F).

A left fronto-temporal craniotomy, with interfascial dissection of the temporalis muscle and the harvesting of a pedicled pericranial flap, was performed. EEG, MEP, SSEP, and EMG of the left CN III and VI were used for the surgical excision of the lesion.

After the removal of the hyperostotic and exophytic components below the temporalis muscle, the fronto-temporal dura was exposed, and the pre-operatively planned cutting guide was used during this phase to model the craniectomy (Figure 3I). The left superolateral periorbita appeared to be invaded by the lesion, and the removal of the intraorbital component of the meningioma allowed for the decompression of the orbit.

After a crescent-shaped dural opening, the excision of the intradural component of the meningioma was performed. A subtotal resection of the lesion was obtained as a tight adherence with the lateral muscle was evident at the level of the intraorbital, subperiosteal component. The pedicled pericranium flap was used to cover the only partially opened frontal sinus. The PEEK custom-made operculum was placed and fixed with plates and screws (Figure 3I) to obtain an optimal cosmetic result.

The histological examination confirmed the diagnosis of meningothelial meningioma (CNS WHO grade I). In the immediate postoperative period, anterior blepharitis and transient paralysis of the temporal branch of the facial nerve were evident. The postoperative CT scan documented the optimal reconstruction without acute complications (Figure 3J–L).

At four months, the follow-up MRI showed that the lateral rectus muscle and the ipsilateral optic nerve had re-expanded compared to the previous pre-op brain MRI. A five-millimeter residue into the orbit contacting the lateral rectus muscle was also identified (Figure 3M–O).

At the latest follow-up, the aesthetic outcome is satisfactory, and complete resolution of the exophthalmos with no enophthalmos is evident.

## 4. Discussion

In this study, we shared our experiences in skull base reconstruction using 3D computer-assisted printing implants to manage spheno-orbital meningiomas that cause marked hyperostosis at the level of the pterional and orbital regions. An optimal therapeutic goal must not only be focused on a satisfactory removal of the lesion and functional recovery of the patient but also on obtaining an excellent aesthetic outcome [12,23,24].

Traditionally, the gold standard for the reconstruction of skull base bone defects is represented by the use of autologous tissues, such as a portion of calvarium, costal, or iliac graft, although these reconstructive techniques are not free from potential complications with considerable discomfort for the patient [3,5,6,7]. Furthermore, reconstruction might be too complex to achieve with a simple graft as the defect can be geometrically complex, involving not only the superior and lateral walls of the orbit but also the pterional region.

Over the last twenty years, technological development has introduced innovative materials to replace autologous tissues capable of adapting perfectly to the patient’s anatomy, depending on the pathology for which reconstruction is required. Nowadays, surgeons have several materials available such as polymethylmethacrylate, methylmethacrylate, titanium mesh, medpor, porus ethylene, or different combinations of multiple materials that can be easily contoured during the surgery to repair a variety of skull defects [3,13,25]. Each material presents advantages and disadvantages that must be considered, such as the ability to integrate with the patient’s anatomy, the radiolucency, the risk of infection, and its costs [15,17,18]; but generally, as they are practical to model according to needs, they are able to offer a less than precise correction of the bony defect. Nonetheless, the difficulty of repairing precisely a geometrically complex region remains.

The necessity and modality of repairing bone defects in orbital roof and lateral wall reconstruction are still debated today [4,7,26,27]. Although it is mandatory to avoid potential postoperative complications, it is not uncommon to document risks associated with reconstructive approaches, including pulsating enophthalmos, meningocele, CSF leak, and meningitis [7,11,26,28].

The introduction of 3D printing patient-specific cranioplasties modeled on pre-operative CT scans has revolutionized the degree of accuracy and precision of the reconstruction phase thanks to computer-aided design (CAD) and manufacturing (CAM) [3,13,15,18]. This technology is opening up new scenarios in the challenge of reconstructing different areas of the skull base [29,30].

In a recent study, Hosameldin et al. highlighted that the functional outcome was significantly higher in the custom-made 3D group compared to the handmade cement bone (60.6% vs. 0%; 21.2% vs. 0%, *p* < 0.05), and that late complications were noted in 50% of the handmade bone group (vs 0%, *p* < 0.05) [31].

Regarding spheno-orbital meningiomas, eleven papers described 27 patients who underwent reconstruction using 3D printing custom-made cranioplasty [3,11,12,13,14,15,16,17,18,19,20]. Most of the papers analyzed in this review described the potential benefits of one-piece reconstruction prostheses, while Korn et al. reported a two-piece patient-specific implant [15]. Most reconstructed the bony defect at the time of tumor removal [3,11,12,13,15,16,17,18,19,20], but some have reported delayed reconstruction, mainly to address the postoperative enophthalmos [3,14].

Although the benefits of these modalities of reconstruction are well known, their short- and long-term complications need to be better reported. Two cases of misaligned implants with subsequent surgical revision were described [13,15], while no cases of infection have been reported either in the literature or in our personal experience in the management of spheno-orbital meningiomas. Therefore, the complexity of repairing the antero-middle skull base bony defect is based on the difficulty of reconstructing the orbital region, avoiding excessive pressure [13]. The filling of the intraorbital space and repairing dural tears with autologous fat, combined with the use of a rigid prosthesis, may cause overpacking, which may require a revision surgery with potential risks to the functionality of the eye [9]. On the other hand, an insufficient filling of the empty volume could cause enophthalmos and facilitate incorrect healing, with CSF leak and meningitis. The creation of cutting guides may support the surgeon in preventing this type of complication [3]; however, certain expertise and surgical experience are required to obtain optimal results, and, in some cases, the bony resection could be greater than previously planned, modifying the degree of adaptation with the patient’s anatomy [12].

Another crucial point to consider is the possibility of correcting any defect of soft tissues, such as the atrophy of the temporalis muscle, by compensating the defect with the use of customized 3D custom-made prostheses, although opinions are conflicting [12,13]. In our series, this was not necessary. All of our patients were satisfied with the final aesthetic outcome without any deformities; certainly, all of them had been counseled pre-operatively on the challenges of normalizing a condition that was markedly abnormal and of the postoperative changes that usually take place up to one year after surgery. Overall, the literature generally reported satisfactory cosmetic outcomes. Among the cases reviewed, four patients exhibited facial asymmetry with minor deformities [3,11,12]. Additionally, one patient had a notable preoperative facial deformity, which was improved but still present to a lesser extent, during the postoperative period [14]. One patient had a suboptimal outcome with visible deformity [13]. However, the data available in the literature are limited and poorly focused on this aspect, which has a significant impact on patients’ quality of life.

Further studies would be necessary to systematically analyze the aesthetic results of patients treated with different reconstruction methods based on the degree of patient satisfaction in order to quantify the superiority of different types of customized 3D prostheses compared to other reconstruction techniques.

Finally, regarding the average production cost, 3D printing custom-made is more expensive (3500–4500 E) than other reconstructive materials such as titanium mesh, medpor, or formable thermoplastic polymers, yet it seems to offer superior results in terms of aesthetic outcome [6], especially in cases of large bony defects that are geometrically complex, as they involve the pterional regional, as well as the orbit. In recent years, the realization of less expensive materials has allowed for an initial diffusion of this reconstruction technique in malignant pathologies of the skull base, such as epidermoid carcinoma or metastasis [32,33], highlighting the importance of offering a better quality of life event to patients with an unfavorable outcome.

## 5. Limitations of the Study

This retrospective study included a small sample size of patients, which may influence the quality of the research and increase the risks of biased interpretation of the data, especially for postoperative complications (infection, misaligned prosthesis, CSF leak, pulsating enophthalmos, and need for re-operation), and cosmetic outcome.

Regarding the literature review, the authors only considered papers written in English and documented cases of patients affected by spheno-orbital meningiomas. Therefore, all cases of 3D printing reconstruction of the same anatomical region for the treatment of other pathologies were excluded.

## 6. Conclusions

Our preliminary experience using custom-made 3D-printed cranioplasty to treat spheno-orbital meningiomas has led to encouraging results, with a high degree of functional and aesthetic satisfaction for our patients.

However, further clinical studies with larger cohorts are mandatory to evaluate the potential benefits of this reconstructive technique and discover further applications, especially regarding long-term cosmetic outcomes, durability, type of implants, and rate of infection.

Technological innovation in biocompatible materials and their processing could facilitate the development of low-cost technologies so that they can be used more widely.

## Figures and Tables

**Table 1:** Summary of the data reported in the included publications.

First Author, Year of Publication	Age (Mean; Range)	Sex (%)	MAT (%)	Surgical Approach (%)	Steps (m ^§^)	Pre-op. Symptoms	Resection	Post-op. Neurology	Complications (%)	Outcome (mRS) [FU-mo]	Cosmetic Outcome	RT
Pritz, 2009 [11]	52	F	PMMA	FT	1	Vis. I, V1 Medial rectus dysfunction	NT	Intact	N	1 [1.2]	3	NR
	33	F	PMMA	FT	1	Anis, Ex	ST	D	N	1 [1.2]	-	NR
Gerbino, 2013 [17]	54	F	PEEK	FT	1	Dyst, Ex	NR	tD	N	0 [24]	1	N
	56	M	PEEK	FT	1	Vis. I, D, Ex	NR	tD	N	0 [24]	1	N
Schebesch, 2013 [16]	40	F	PT	FT ^	1	Sw, Ex	NT	NR	N	NR	-	NR
	64	F	PT	FT ^	1	Temp. Sw	NT	NR	N	NR	-	NR
Jalbert, 2014 [18]	46	F	PEEK	FT	1	Ex	NR	Ptosis, scalp HyperE	N	2 [12]	1	N
Carolus, 2017 [12]	43	F	PT	FT	1	NR	NR	NR	N	0 [6]	3	N
	64	F	PT	FT	1	Ex	ST	NR	N	1 [6]	3	Y
Bachelet, 2018 [3]	52	F	PT	SC	2 (19)	Eno, D	NR	Intact	N	NR	1	NR
	42	F	PT	SC	2 (24)	Eno, D	NR	Intact	N	NR	1	NR ^ç^
	49	F	PT	TP	2 (22)	Eno, D	NR	D	Eno, SubO position	NR	3	NR
Bassi, 2020 [19]	NR	NR	PMMA	FT	1	Ex, Dyst	NR	Intact	N	0 [36]	-	NR
	NR	NR	PMMA	FT	1	Ex, Dyst	NR	Intact	N	0 [32]	-	NR
	NR	NR	PMMA	FT	1	Ex, Dyst, Vis. I	NR	Intact	N	0 [30]	-	NR
Goertz, 2020 [13]	63	F	PT	FT	1	Ex, Dyst, Vis. I	ST	Intact	Epidural	1 [17]	1	Y
	54	F	PMMA	FT	1	Ex	NR	Intact	SubO position	2 [25]	4	N
	46	F	PMMA	FT	1	Ex, D, Vis. I	ST	Vis. I	N	1 [18]	1	Y
Laroche, 2022 [14]	39	F	PT	FT + OEx	2 (12)	Ex, no functional eye	NT	Intact	N	2 [36]	4	Y
D’Avella, 2023 [20]	70	F	PMMA	FT + TOE	1	Ex	ST	tD	N	0 [3]	1	NR
Korn, 2023 [15] *	(56; 41–89)	F (70)	PT (100)	FT (100)	1	D (70%)	NR	NR	N	0–1 * [NR] (100)		NR

Abbreviations: Anis, Anisocoria; D, Diplopia; Dyst, orbital Dystopia; Eno, Enophthalmos; Ex, Exophthalmos; FT, Frontotemporal; FU: Follow Up; HyperE, Hyperesthesia; MAT, Material used for reconstruction; N: No; NR, Not Reported; NT, Near Total Resection; OEx, Orbital Exenteration; PEEK, Polyetheretherketone; PMMA, Polymethylmetacrylate; PT, Porous titanium; RT, Radiotherapy; SC: subciliary; t, transient; SubO, SubOptimal; ST, Subtotal Resection; Sw, swelling; Temp., Temporal; TP, Transpalpebral Approach; TOE, Transorbital Endoscopic; Vis. I, Visual Impairment; V1, Hypoesthesia on the first trigeminal branch territory; Y, Yes. * The series by Korn et al. [15] reports aggregated data for 7 patients with two-piece reconstruction; mean values are reported. ^§^ Reports the month when a second staged reconstruction was performed. ^ç^ Implant removed after 7 months due to disease recurrence. ^ no apparent reconstruction of the orbit.

**Table 2:** Details of the four patients who underwent surgical excision of spheno-orbital meningioma and one-step 3D-printed cranioplasty.

Patient	Age	Sex	MAT	Surgical Approach	Steps	Pre-op. Symptoms	Resection	Post-op. Neurology	Complications	Outcome (mRS) [FU-mo]	Cosmetic Outcome	RT
1	60	M	PEEK	FT	1	Ex, D, Ptosis	NT	tVII bleph	N	0 [9]	1	N
2	58	F	PEEK	FT	1	Ex, Vis. I Conj Hyp	ST	Intact	N	0 [19]	1	N
3	63	F	PEEK	FT	1	Ex	ST	Intact	N	0 [32]	1	N
4	53	F	PEEK	FT	1	Ex, Vis. I, D	NT	III, tV2	N	1 [41]	1	N

Abbreviations: bleph, Anterior Blepharitis; Conj Hyp, Conjunctival hyperaemia; D, Diplopia; Ex., Exophthalmos; FT, Frontotemporal; FU, Follow Up; MAT, Material used for reconstruction; N: no; NT, Near Total Resection; PEEK, Polyetheretherketone; RT, Radiotherapy; ST, Subtotal Resection; t, transient; Vis. I, Visual Impairment; III, persistent partial third cranial nerve deficit; tVII, transient deficit of the temporal branch of the facial nerve; tV2, transient hypoesthesia in V2 branch.

## Data Availability

The data presented in this study are available on request from the corresponding author.
